# The Affective Science Network: A Fieldwide Map of over 1 Million Citations

**DOI:** 10.1007/s42761-024-00292-8

**Published:** 2025-02-01

**Authors:** Alessia Iancarelli, Nicholas R. Rypkema, Maureen Ritchey, Ajay B. Satpute

**Affiliations:** 1 Department of Psychology, Northeastern University, 360 Huntington Ave, 125 NI, Boston, MA 02115 USA; 2 Applied Ocean Physics & Engineering, Woods Hole Oceanographic Institution, Woods Hole Rd, Woods Hole, MA 02543 USA; 3 Department of Psychology and Neuroscience, Boston College, Chestnut Hill, MA 02467 USA; 4 Department of Psychology, Core Faculty, Institute for Experiential Artificial Intelligence, Northeastern University, Boston, MA 02115 USA; 5 Department of Radiology, Massachusetts General Hospital, Boston, MA 02114 USA

**Keywords:** Citation network analysis, Affective science, Emotion, History, Bibliometrics

## Abstract

Research in affective science includes over one hundred thousand articles, the vast majority of which have been published in only the past two decades. The size and rapid growth of this field have led to unique challenges for the twenty-first-century scientist including how to develop both breadth and depth of scholarship, curb siloing and promote integrative and interdisciplinary framework, and represent and monitor the field in its entirety. Here, we help address these issues by compactly mapping out this enormous field using citation network analysis (CNA). We generated a citation matrix of over 100,000 publications and over 1 million citations since the seminal works on emotion by Charles Darwin (1872) and William James (1884). Using graph theory metric and content analysis of titles and abstracts, we identified and characterized the contents of 69 research communities, their most influential articles, and their interconnectedness with each other. We further identified potential “missed connections” between communities that share similar content but do not have strong citation-based connections. In doing so, we establish the first, low-dimensional representation, or field-wide map, of a substantial portion of the affective sciences literature. This panoramic view of the field provides affective and non-affective scientists alike with the means to rapidly survey dozens of major research communities and topics in the field, guide scholarship development, and identify gaps and connections for developing an integrative science.

Affective science is undergoing an extraordinary expansion propelled by a nexus of theoretical, methodological, and institutional developments (Damasio, 1994; Dukes et al., 2021; Gross, 2013). This “explosion of affective science” (Fox, 2018; Sander & Scherer, 2014), as it has been called by researchers, now includes hundreds of thousands of scientific articles in the field, the overwhelming majority of which were published just in the past decade. While a few decades ago it was conceivable that dedicated scholars could have a grasp of the field in its entirety, today, this notion is far beyond the capacity of a human being. This era of remarkable growth brings forth several new challenges to the field. How are we to represent the total accumulation of scientific knowledge in affective science or any other large-volume scientific discipline? What does it mean to develop expertise in a field, both in terms of breadth and depth? And how can we advance research programs that enable interdisciplinarity while guarding against the natural tendency toward siloing?

Here, we aim to provide the first, large-scale network map of a representative portion of the affective science literature. To be useful, such a map should provide an information-rich but compact (low-dimensional) representation of the literature that characterizes affective science in terms of the number, size, content, and intercommunication of its subfields. We approximate this structure using citation network analysis (CNA) (Bruner et al., 2013; Moore et al., 2005), which analyzes the patterns of citations between papers to identify research communities. CNA in fields as large as affective science has only recently been made possible due to advances in bibliometrics and information processing algorithms. We further combine CNA with content analysis (e.g., of titles, abstracts, keywords) to determine the topics and most influential papers of each community. The resulting “affective science network” provides a panoramic view of the field with the capability to identify potential missed connections between subfields to help curb siloing, and a roadmap to efficiently develop both breadth and depth of scholarship.

## The Research Explosion of Affective Science

**Fig. 1:**
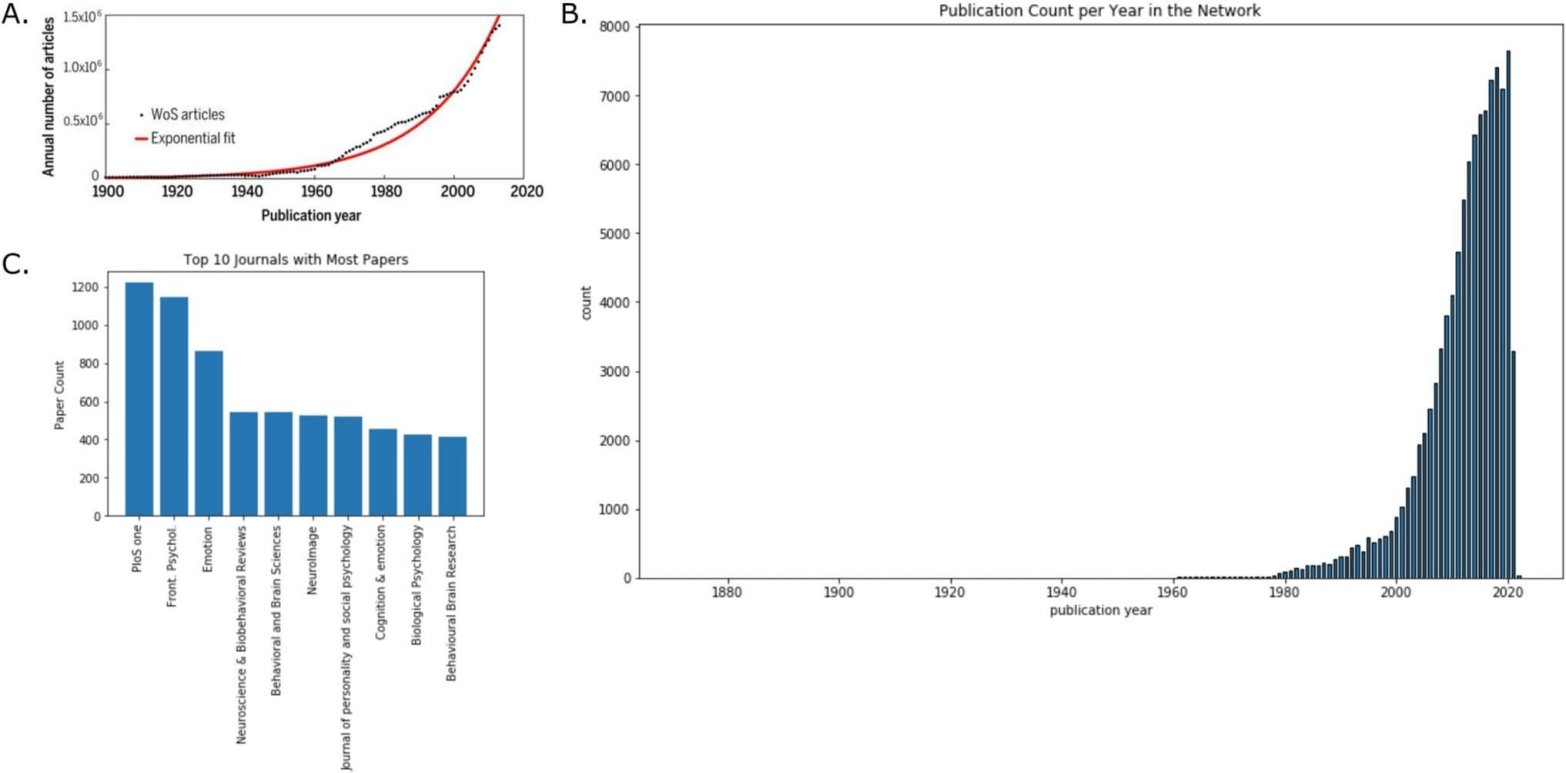
**A** The number of scientific publications follows an exponential trend with an average doubling rate of every 15 years (copied from, Fortunato et al., 2018; copyright permissions obtained via email). **B** The distribution of publication years for the papers in our network is also exponential. Mean = 2,011.97; median = 2,014; mode = 2,020; variance = 61.37; *SD* = 7.83. Only .07% of papers lack a publication year. The 2021 drop is likely due to incomplete 2022 data. **C** The most frequent publication outlets for papers in our citation matrix database

The accumulation of scientific literature is thought to follow the law of exponential growth (de Solla Price, 1976) with an average doubling period of 15 years (Fortunato et al., 2018) (Fig. 1A). Research in affective science, as represented in our network, follows suit (Fig. 1B). In the past 50 years, the rate of publications in affective science skyrocketed from less than 1,400 article publications per year to nearly 175,000 publications in 2022 alone. This figure, though large, is consistent with the trend in psychology more broadly. PSYCINFO, which has been cataloging articles since 1927, announced its 4 millionth record nearly a decade ago (press release, 2015). As described below, several factors have enabled or directly contributed to, this massive accumulation of work (Dukes et al., 2021; Gross, 1998).

### Empirical and Methodological Advances in Affective Science

A key factor contributing to the “rise of affectivism” (Dukes et al., 2021) is the growing recognition that affective processes are both ubiquitous and inescapable. Centuries before the birth of the first experimental psychology research lab in the late 1870s, emotions and feelings were a central focus of religion, philosophy, politics, and more (Firth-Godbehere, 2021; Rosenwein & Cristiani, 2017; Solomon, 1993b). However, it took a handful of empirical demonstrations to show that affective processes are also inescapable (LeDoux, 1998). Early thinking in affective science, likely influenced by Western philosophical traditions (Descartes, 2016; Plato et al., 2008), assumed a distinction between “thinking” and “feeling” with the hopes that sound judgment followed the former and not the latter. This idea was upended with empirical demonstrations showing that feelings not only infiltrate our decisions (Zajonc, 1980) but also may be necessary for them to be made in the first place (Damasio, 1994).

The 1960s marked the emergence of a multidisciplinary interest in affective processes leading to a substantial expansion of the field. Part of this growth is attributable to theoretical inspirations from the New Look movement (Bruner, 1957; Bruner & Goodman, 1947) and cognitivist approaches (Barrett & Russell, 2014; Russell, 2003; Schachter & Singer, 1962), which laid the groundwork for the idea that virtually no mental faculty was impervious to affective influence. Empirical demonstrations soon followed showing that affective processes contribute to a wide variety of phenomena including the sensation of perception (Villemure & Bushnell, 2002), learning and memory (Tyng et al., 2017), social cognition (Blanchard-Fields, 1997; Sander et al., 2005), and reasoning (Blanchette & Richards, 2010). These ideas subsequently spread to several disciplines outside of laboratory research. Indeed, affective processes have been shown to influence whether and how doctors make diagnoses (Estrada et al., 1997; Liu et al., 2022) judges and juries make rulings and verdicts (Vasilyev, 2017; Wistrich & Rachlinski, 2017), and people decide to engage in political action (Marcus, 2000).

In parallel, methodological advances have led to entirely new areas of research in affective science. Since their origins in the late nineteenth to early twentieth centuries, scientific methods to measure and manipulate subjective emotion (Young, 2018), facial behavior (Landis, 1924), and autonomic activity (Cannon, 1914) have undergone major refinements due to innovations in experimental methods and the capacity for electronic recordings of behavior (AlGhatrif & Lindsay, 2012; Barrett & Barrett, 2001; Ekman & Friesen, 1978; Young, 2018) and in computing and statistical analysis (Azari et al., 2020; Snoek et al., 2023). In recent decades, recordings of brain activity (e.g., EEG, PET, and fMRI) have led to several new areas of research spanning fundamental questions about the biological nature of emotions (Kragel & LaBar, 2016; Lindquist et al., 2013; Mobbs et al., 2019) to applied directions for biofeedback applications (e.g., (Sitaram et al., 2007) and affective robotics or virtual agents (Marsella et al., 2010). The combination of empirical insights and methodological innovations is likely to have spurred several subfields in affective science (Dukes et al., 2021). Here, citation network analysis may discover how many of these communities there are, their topics, and the extent to which they communicate with one another.

### Blossoming of the Professional Landscape

The professional landscape for affective science has also undergone significant transformations which have contributed to the volume of scientific publications (Fortunato et al., 2018). There are now an increasing number of research centers, journals, and societies that are exclusively dedicated to affective science or subfields therein (Dukes et al., 2021). The past two decades alone saw the birth of at least four new major societies: the Social and Affective Neuroscience Society (SANS, est. 2007), the Society for Affective Science (SAS, est. 2014), the European Society for Cognitive and Affective Neuroscience (ESCAN, est. 2009), and the Australasian Society for Social and Affective Neuroscience (ASSAN, est. 2014). These organizations parallel two others that have been established since the 1980s including the International Society for Research on Emotion (est.1984) and the Consortium of European Research on Emotion (est. 1986). In earlier decades, the limited number of publishing outlets may have created a bottleneck effect (and perhaps more unpublished works), but this has changed with the launch of field-specialized journals including *Cognition and Emotion* (est. 1987), *Emotion* (est. 2001), *Social Cognitive and Affective Neuroscience* (est. 2006), and most recently, *Affective Science* (est. 2021). Perhaps more strikingly, however, is the impact of newly formed journals: *PLOS ONE* (est. 2006) and *Frontiers in Psychology* (est. 2010). Based on our dataset, counting over 100,000 publications, these two journals account for far more work published than many of the more established outlets combined (Fig. 1C). Collectively these changes to the field’s professional landscape have played a tremendous role in enabling a large volume of publications in the field.

### Reframing Research Priorities

Research funding may also have led to a growing interest in affective science by scientists across areas of psychology. In 2009, the National Institute of Mental Health (NIMH) initiated the Research Domain Criteria (RDoC) project, a pioneering effort aimed at the comprehensive classification and organization of mental illnesses (Insel et al., 2010). The advent of RDoC led to a paradigm shift by organizing research objectives into five core domains, three of which directly pertain to affective science including negative valence systems, positive valence systems, and arousal/modulatory systems. In 2018, the National Institutes of Health (NIH) further allocated funding to support the establishment of dedicated research networks designed to address emotional well-being.

## Challenges for Twenty-first Century Science

### Quantity and Quality

The volume and acceleration of research output have led to unique considerations for twenty-first-century scientists. Acceleration of publications may be indicative of maturity (e.g., of methodology and research skills training) or of a quickening pace of new discoveries in a field (Price, 1965). However, and perhaps owing to the commodification of science (Radder, 2010), there are also serious concerns about both the reproducibility of findings (Baker, 2015) and faked results (Sabel et al., 2023). The growing competition for academic positions has also intensified the “publish or perish” mentality in early career scientists (Becker & Lukka, 2023; Rawat & Meena, 2014). Indeed, assistant professors hired in the 2012–2016 cohort had 57% more published articles compared to the 2006–2011 cohort (Pennycook & Thompson, 2018). This trend has now filtered down even to high school students who are lured by costly services that help them obtain first-author journal publications (ProPublica, 2022). There have been calls to stymie the overbearing influence of publications over assessing the quality of the work or the merit of the scientists involved (Vale, 2012).

### Scholarship Development

Barring any major systemic changes to the field, the fact remains that even today, the volume and acceleration of research output far exceeds the ability of any scholar to remain on top of it. Without the capacity to read each and every article in a field, trainees must lean heavily on narrative reviews or books, guidance from a mentor, and conduct their own searches. Prior to the internet, scholarship was developed by laboriously tracing backward through reference sections or by reading titles of journal articles from tables of contents. Internet search engines such as PubMed and PsycINFO dramatically reduced the burdens of identifying relevant works. However, research volume has still outpaced the ability to consume it. It is common to hear researchers lamenting the fact that they had hundreds if not thousands of email alerts of journal issues that have gone unread. In response to volume, search criteria for email are also likely to have shifted in a worrisome way from broader, topical searches (e.g., using PubMed topic alerts) to only when articles are published by specific, well-known principal investigators (a strategy that would exacerbate citation biases in the field, a topic we discuss below). Some research labs have adjusted by distributing the burden of developing field expertise across lab members. For example, students from different labs would be responsible for reviewing and summarizing key articles from select journals. But this strategy, too, has been unable to address the tide of publications. Students and researchers today may be abandoning the notion of comprehensive scholarship in favor of focusing on what is popular or trendy. To point out, many students have indicated that they rely on social media feeds such as X (formerly Twitter) to announce which articles may be of interest to the user (Ke et al., 2017; Lulic & Kovic, 2013). It stands to reason that these issues may lead to discouragement that field expertise is no longer within reach coupled with anxiety or imposter syndrome (Jaremka et al., 2020) from a pervasive sense of insufficient scholarship.

### Siloing

The acceleration of research publications may increase siloing wherein researchers in a community become increasingly isolated from other communities in the field even when they are studying conceptually overlapping phenomena. Siloing can have several consequences. First, it may restrict the flow of ideas and innovations across subfields, preventing researchers from benefiting from advancements in related areas and from developing an interdisciplinary understanding of affective phenomena (Schiller et al., 2024). Second, it can lead to confusion between research communities. For instance, two students who are interested in the science of “fear” may learn completely different ideas about what fear is, how to study it, and how to interpret the findings, such that they are confused by one another’s scientific approach when in truth the ontological presuppositions that guide research (and which should be used to evaluate research from their respective frameworks) are vastly different (Barrett, 2022; Dubova & Goldstone, 2023). Third, siloing may contribute to redundant research efforts, as researchers in different subfields may unknowingly address similar problems or develop overlapping solutions without being aware of each other’s work. Siloing may be particularly concerning in fundamental v. translational (Gross & Jazaieri, 2014, p. 20; Larsen & Hastings, 2018) research areas. For example, a notable discrepancy can be observed in the research landscape, where the number of studies examining the interconnections between affective and psychiatric phenomena, such as fear and panic attacks, is considerably smaller compared to studies that investigate these phenomena as separate, isolated categories (Gross & Jazaieri, 2014; Larsen & Hastings, 2018). However, the extent to which research areas are at risk of silofication remains unclear. While CNA offers a valuable method for identifying subfields within the network, caution should be taken when interpreting citation-based communities as purely reflective of ontological factors. Thus, it is important to acknowledge that sociological factors such as shared tools, conventions, lab ancestry, and geographic proximity may also contribute to the separation of communities detected by CNA (Bird, 2013; Leonelli, 2016). Here, CNA may provide data-driven insights for identifying whether research publications surrounding a topic are indeed divided into siloed research communities.

### Citation Bias

Intermingled with concerns over innovation and the development of scholarship expertise is citation bias with respect to field-wide demographics. These are now being quantified and exposed. For example, white men are more likely to be cited than their peers from underrepresented groups even when controlling for many potential confounding factors (e.g., (Teich et al., 2022). Reference lists tend to include more papers with a white person as the first and last author than would be expected if race and ethnicity were unrelated to referencing (Bertolero et al., 2020). This imbalance is driven in part by the citation practices of white authors and, strikingly, is increasing over time, even as the field diversifies (Bertolero et al., 2020). The acceleration of research volume might be a contributing factor to citation bias as researchers must increasingly rely on heuristics to develop their knowledge base.

CNA can recapitulate these very same issues since it inherently relies on the citation practices of scientists (Dworkin et al., 2020). Yet, CNA is not a monolithic approach; rather, it refers to a family of flexible approaches that may help scientists enact more balanced citation practices. A prior CNA of research on aggression (Iancarelli et al., 2022) identified the citation-based influence of articles in two ways, first by using a more traditional count of citations for the entire aggression field and second by first organizing the field into citation-based communities and then using outdegree measures per community. Iancarelli et al. (2022) found that the number of articles labeled as influential with first authors who are women depended on the method; it doubled when conditioning citation count by research communities. Moreover, this CNA approach provided researchers with the means to easily identify these articles, and thus, to engage in more balanced citation practices.

## Generating an Affective Science Network

Barring any major structural or institutional changes (e.g., some have suggested imposing a ceiling on the number of publications per research lab), one key step forward to address the acceleration of research is the develop analytic strategies that can provide an overview of scientific literature in its entirety using compact (e.g., 2-D, visualizable), but information-rich representations. Existing online tools can facilitate visualization of connections among papers based on their citation practices (e.g., Connected Papers (Connectedpapers.Com, accessed 2024), ResearchRabbit (Researchrabbit.Ai, accessed 2024)), but these tools are optimized for paper discovery, not for surveying an entire research field. A map of the scientific literature may help reduce the burdens of developing expertise and concomitant barriers to entry for newcomers to the field by providing information about which research communities exist, what their topics are, and which articles have had a strong impact on those communities. It may also help overcome redundancies, communication barriers between fields, and missed opportunities for interdisciplinary collaborations owing to siloing, all of which may contribute to improving the overall quality of scientific research.

Using a combination of citation network analysis (CNA) and content analysis, we generated an affective science network—a network organization of the affective science literature including its structure as a set of subfields, the contents of these subfields, and their interconnections. Affective science encompasses a wide variety of topics that already have several books devoted to them including on the fundamental nature of emotion (Barrett, 2017; Damasio, 2006; Prinz, 2004), emotion perception (e.g., from faces (Ekman, 2006; Russell & Fernandez-Dols, 1997)), affective neuroscience (LeDoux, 1998; Panksepp, 2004), emotion measurement and methods (Coan & Allen, 2007), decision-making and emotion (Rolls, 2013; Vohs et al., 2007), emotion and psychopathology (Flack & Laird, 1998), and more (Davidson et al., 2009; Ekman & Davidson, 1994; Gross, 2013). Such topics or subtopics therein are also discussed in literature reviews, and it stands to reason that our CNA will recover similar topics amongst communities accordingly. Surpassing prior reviews, the affective science network also provides a data-driven map that can be used to characterize the field in its entirety. This map can further be used to (a) identify silos by quantitatively identifying communities with little connection to other communities, (b) forge connections between communities that are distant in terms of citations but otherwise share similar topics, and (c) guide both depth and breadth of scholarship development by providing topical descriptions of each community, the most central papers within each community, and both bibliometrically and topically organized pathways to other relevant research communities.

## Methodology

### Overview

CNA emerged during the latter half of the twentieth century, finding its initial application in the analysis of the historical events described by the author Isaac Asimov in his book *The Genetic Code* (Garfield et al., 1964). It was first used to quantitatively map the structure of a research field a decade later (Small, 1973). However, there have been certain barriers that prevented CNA from being deployed in science more widely. Limitations in bibliometric databases (i.e., public access and breadth of work), computing power, and computational algorithms have made it difficult if not impossible to model a field as vast and complex as affective science. Only recently has this collective set of limitations been surpassed for the purposes of literature review of relatively small fields (Benckendorff & Zehrer, 2013; Dawson et al., 2014; Gustafsson et al., 2014; Iancarelli et al., 2022; Leng & Leng, 2021), and meta-scientific questions (e.g., pertaining to citation bias, favoring positive results) (Duyx et al., 2017; Fortunato et al., 2018).

Here, we obtained a citation database involving over a million citations from the affective science literature. Given the massive size of the citation matrix (i.e., > 1 terabit), we used the recently developed Leiden community detection algorithm to estimate the community structure within this matrix. We then used a combination of graph analyses and content analysis to characterize the content and topics of each community and the interrelationships between communities. The resulting affective science network enabled us to generate information-rich data representations of each affective science community and also of the field in its entirety. All analyses were performed using the Discovery Cluster, a high-performance computing resource located at Northeastern University, and using Python (Van Rossum & Drake, 2009) as the primary programming language.

**Fig. 2:**
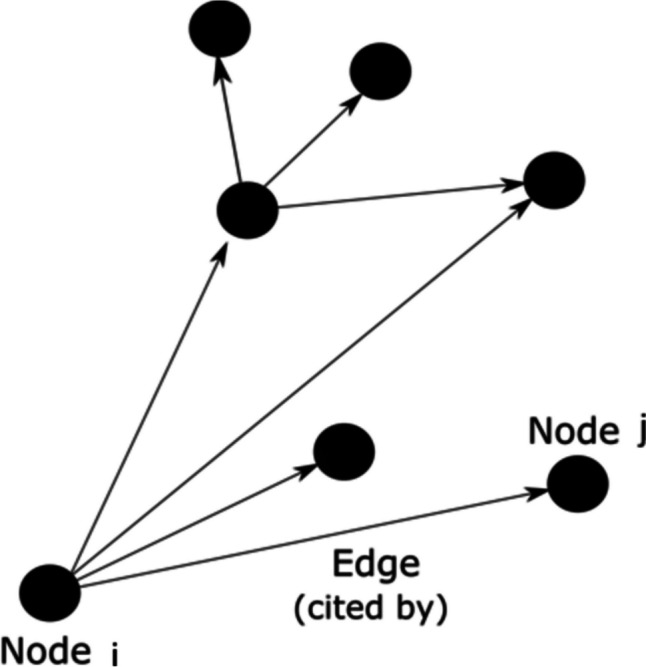
Nomenclature of a small citation network. A node represents a paper in the citation matrix, while an edge (the link between nodes) represents a citation (“cited by”)

In graph theory, a network is composed of “nodes” and “edges” that link nodes. In our CNA, each node refers to a paper and each edge refers to the citation between two papers. Because citations are rarely bidirectional, CNA typically involves directed graphs. For example, and as illustrated in Fig. 2, the arrow pointing from node A to node B indicates that paper A is cited by paper B, or more casually, that node A “influences” node B.

### Citation Database

An important development toward conducting large-scale CNAs is the innovation of computerized academic research databases that collate publications since the very beginnings of psychological science (e.g., PsycInfo, est. 1967; PubMed, est. 1996; Semantic Scholar, est. 2015). For our analysis, we used an application programming interface (API) provided by the free platform Semantic Scholar to gather information on papers including titles, abstracts, citation counts, authors, and publication dates. Commonly used search strategies for developing a citation database include keyword and seed-based searches. Each strategy offers advantages but also limitations (for a discussion, see Baker, 1990 (Baker, 1990).

Here, we used a seed-based approach for a few reasons. First, we are interested in charting out the territory of affective science in the modern era, rather than how emotions and feelings have been discussed in philosophy, religion, literary studies, or other disciplines since antiquity. Second, the history of affective science points to two landmark works that were published at the very beginnings of psychological science and that have directly or indirectly shaped the entire field of affective science that came after it, namely, *The Expression of the Emotions in Man and Animals* (Darwin, 1872) and *What is an emotion?* (James, 1884). These articles have been cited by scholars from a wide range of scientific disciplines including psychology, biology, neuroscience, anthropology, sociology, and philosophy, and their profound influence has been noted in many historical perspectives in these fields (Barnes, 1960; LeDoux & Hofmann, 2018; Mayer, 2011; McDermott, 2004; Ogburn, 1937; Randall, 1961; Solomon, 1993a; Uvnäs-Moberg et al., 2005). Third, seed-based networks provide an inherent structure to the network since each paper is necessarily linked to every other paper, which can be extremely useful for robust network estimation. A limitation of seed-based approaches is that they may exclude research communities that do not link back to the chosen seeds. However, in the affective science literature, wherein William James (2013) and Charles Darwin (1993) are widely acknowledged to be the two major pillars of modern scientific thought, the benefits of taking a seed-based approach outweigh the limitations. Moreover, we took a double-seeded, two-generation approach to developing our database wherein we obtained all papers that directly cite one or both of these sources (first-generation articles), and all papers that directly cite the first-generation articles (second-generation articles) to cover the field since many relevant articles might not directly cite these two seed articles, thus mitigating some of the limitations of the seed-based approach. We did not impose any additional content-based filters. This more inclusive approach may lead to identifying some communities that are only tangentially related to affective science. However, it also ensures that research groups whose work is relevant to affective science are not overlooked, even if they do not use typical affective science keywords. We filtered out papers without any citations, resulting in 101,240 papers and 1,065,422 citation links between papers.

### Community Detection and Validation

Another important development toward conducting large-scale citation network analysis is the innovation of efficient computational algorithms that can perform graph theoretic analyses on large datasets. To identify community structures in our matrix, we used the recently developed Leiden community detection algorithm (Traag et al., 2019), which is both computationally efficient and ensures well-connected communities. The Leiden algorithm is efficient because, at each step, it makes the locally optimal decision (“greedy algorithm”) to merge two communities if doing so increases the modularity (i.e., the connections within communities are denser than the connections between communities). Thus, the Leiden algorithm ensures well-connected communities by iteratively merging communities that are not well-connected (“refinement”). This process ensures that the communities that are identified are cohesive and interconnected.

We selected the Leiden algorithm because it is designed to handle large networks efficiently while ensuring better-connected and more stable clusters compared to its predecessor, Louvain (Hairol Anuar et al., 2021). The Leiden algorithm optimizes modularity, ensuring cohesive communities and preventing the formation of disconnected communities, which makes it particularly suited for our large-scale citation network of 100,000 papers and 1 million edges. In contrast, flow-based algorithms like Infomap are slow and produce fragmented clusters. Stochastic block models and *k*-means clustering are computationally intensive and require a predefined cluster count, making them unsuitable for our large network and undefined clustering needs.

Given the volume of the literature, and our goal to provide a broad characterization of the literature, we filtered out nodes from very small communities (defined as < 100 nodes) from the results. The Leiden community detection algorithm is a probabilistic clustering algorithm; thus, the obtained solution may depend on initialization, the order of nodes submitted to the algorithm, and the aggregation steps. To evaluate the robustness of the observed community structure, we applied the Leiden algorithm to 200 random subsets of 80% of nodes in our network and recalculated the community structure (Iancarelli et al., 2022). We then used normalized mutual information (NMI), averaged across repetitions, to evaluate the consistency of the results across repetitions (Viola & Wells III, 1997; Niedenthal & Brauer, 2012).

### Community Topics and Content

To characterize the research topics within each community, we employed several methods. First, we extracted all words from the titles, keywords, and abstracts of the articles in each community and submitted them to the Python library “word cloud” (Jin, 2017). This process allowed us to identify the most frequently represented words and phrases (e.g., “emotion regulation”) for each community. Stopwords—words that do not convey meaningful information (e.g., “and,” “a,” “or”)—were automatically filtered out.

We then manually inspected the resulting word clouds and assigned labels to each community, reflecting the most dominant topics. These labels were cross-checked with the ten most influential articles within each community, determined by out-degree centrality, to ensure that the selected labels accurately represented key research topics.

We used several levels of content detail to represent each community’s topics: (i) a concise community label (Table 1), (ii) a set of two or three key phrases (Table 1), (iii) the full word cloud, available on the Affect-Net website, and (iv) the ten most influential articles, also accessible on the Affect-Net website. While the labels in Table 1 offer a brief summary of the dominant topics, they do not capture the full complexity of each community, as demonstrated by the word clouds.

**Table 1:** Community number, topic, and number of papers per community

*N*	Label	Topic	Size
1	Emotion theory	Emotion, facial expression, affect	19,873
2	Emotion regulation	Emotion regulation, depression, children	13,792
3	Empathy	Empathy, social, children	6,945
4	Emotional intelligence	Emotional intelligence, employees, Labor	6,479
5	Disgust	Disgust, women and men, behavior	5,601
6	Reward	Reward effect, addiction, rat	5,516
7	Personality	Personality, animal behavior, social	3,518
8	Facial expression	Facial expression, expression recognition, emotion recognition	3,492
9	Amygdala	Amygdala, rat, mice, anxiety	2,662
10	Infant	Attachment, infant, child, mother, development	1,814
11	Robot	Robot, anthropomorphism, agent	1,527
12	Shame	Shame, guilt, embarrassment, social	1,490
13	Social	Body, social, health	1,468
14	Vagus nerve	Treatment, vagus nerve, gut microbiota, gut-brain	1,349
15	Sentiment analysis	Sentiment analysis, text, emotions	1,074
16	Happiness	Happiness, positive emotions, wellness	818
17	Anger	Anger, aggression, trait anger	744
18	Face	Emotions, right hemisphere, facial expression	723
19	Socio-political	Emotion, political science, social, work	691
20	Pain	Pain, children, pain facial expression	635
21	Political	Political, information, citizen	616
22	Communication	Relationships, communication, communication style	596
23	Behavior	Learning, learning theory, behavioral psychology	585
24	Self	Self, emotion, embodiment, social	515
25	Consumer	Impulse buying, consumer	485
26	Pet	Pet, dog, human relationship	426
27	Game	Game, experience, enjoyment	381
28	Expressive writing	Expressive writing	327
29	Risk	Risk, decision-making, ambiguity	316
30	Method (children)	Children play, social, methods	309
31	Sexual arousal	Sexual arousal, women, men, sexual response	305
32	Experience	Experience, phenomenological analysis	301
33	Humor	Humor, laughter	297
34	Smile	Smile, esthetic, dental	295
35	Biodiversity	Conservation, biodiversity, value, nature	289
36	Odor	Odor, emotion, olfactory	286
37	Student learning	Student learning, teaching, emotion	285
38	Expectation	Expectation, hypnosis, experience	280
39	Design	Design, user experience, body, interaction	279
40	Language	Sign language, language, linguistic	269
41	Depression	Depression, patient behavior, treatment	258
42	Fear	Fear, behavior, depression, infant	245
43	Creativity	Creativity	239
44	Agent	Agent, simulation, emotion model, system	232
45	Stress	Depression, stress, sex differences	194
46	Time	Time perception, duration	194
47	Biological	Biological, functions, traits	190
48	Tourism	Tourism, cultural	187
49	Play	Children play, intervention	186
50	Asthma	Asthma, anxiety, children, autism, ADHD	185
51	Crying	Crying, tear, emotion	180
52	Mind	Emotion, mind, consciousness, experience	179
53	Dark tourism	Dark tourism, tourist	170
54	Interaction (user)	Interaction, game, design, user, interface	160
55	Women	Women, media, body image	159
56	Student learning II	Student learning, performance	150
57	Animal behavior	Animal behavior, learning	146
58	Attitude	Attitude, behavior	145
59	Nostalgia	Nostalgia	145
60	Temperament	Temperament, adolescence, parent, social	144
61	Media	Political opinion, media, communication	139
62	Culture	Culture, self, collectivism, individualism	136
63	Measure	Construct, measure, marketing	136
64	Depression II	Depression, anxiety, disease	135
65	Action	Action, response, action effect	125
66	Experience (music)	Learning, music, experience, intervention	108
67	Speech	Speech, model, user, learning, system	101
68	Competence	Competence, patient consent, capacity, decision-making	101
69	Soil	Soil, earthworm, sympathy	101

The table presents a brief summary of the dominant topics, without fully capturing the complexity of each community (as shown in the word clouds available on Affect Net). The Leiden algorithm clustered 91.5% of the papers into 69 communities containing at least 100 papers. The remaining 8.5% of papers formed thousands of communities containing only a few papers each, many of which were constituted by single papers (*N* = 4,922) and were filtered from further analysis

$$C(v) = E(v) / (N-1)$$

Formula 1 (out-degree centrality), where *C*(*v*) is the out-degree centrality of node *v*, *E*(*v*) is the number of outgoing edges from node *v*, and *N* is the total number of nodes in the network.

### Community Interconnectedness

We also characterized each community in terms of how it communicated with other communities in terms of citations. Here, we used a strategy employed by Iancarelli et al. (2022) wherein the citation relationship between two communities of nodes is defined by the average of the shortest path links between nodes of each community. Given the size of the network, it is computationally costly to calculate the shortest path links between all nodes of pairwise communities. Thus, we first used betweenness centrality scores (Formula 2) to select the top 10% of papers, per community. By definition, these are the papers that are the most interconnected with the rest of the network and thus are useful for curbing the computational cost of identifying the shortest path lengths between communities.

$$B(v)=\Sigma (s\ne v\ne t)\sigma (s,t|v)/\sigma (s,t)$$

Formula 2 (betweenness centrality). *B*(*v*) is the betweenness centrality of node *v*, *σ*(*s*, *t*) is the total number of shortest paths between nodes *s* and *t*, and *σ*(*s*, *t* | *v*) is the number of shortest paths between nodes *s* and *t* that pass through *v*. The betweenness centrality of a node is thus the sum of the fraction of all shortest paths that pass through that node (throughout the entire network).

We then defined the interconnection between communities as the average shortest path length between the top 10% of most central papers from each pair of communities. This method helps us identify the most efficient routes for “information flow” in terms of citations between communities (Formula 3).

$$L(A,B)=\frac{{\sum }_{s\in A,t\in B,s\ne t} d(s,t)}{\left|A\left|\cdot \right|B\right|}$$

Formula 3 (average shortest path length between two communities). *L*(*A*,*B*) represents the average shortest path length between communities *A* and *B*, where *A* and *B* are the sets of the top 10% of most central papers from each community. *d*(*s*,*t*) is the shortest path distance between node s ∈ A (from community *A*) and node t ∈ B (from community *B*). The formula sums the shortest path distances between every pair of nodes *s* and *t* from the top 10% of most central papers in their respective communities and averages these distances by dividing by the product of the sizes of the sets *A* and *B*.

### Identifying Missed Connections

We defined a “missed connection” between communities as cases wherein two communities have similar content but low citation-based interconnectedness (as defined above). To calculate content similarity between communities, we used the Python library Word2Vec. Specifically, we employed a custom function “clean_text” to preprocess the titles and abstracts, ensuring that the text is normalized for effective natural language processing. Subsequently, a subset of papers (10%) from each community was randomly selected to create a manageable dataset. Then we trained a Word2Vec model on tokenized text data to generate word embeddings, which capture the contextual usage of words in a high-dimensional vector space. For analytical purposes, document vectors are created by averaging the Word2Vec embeddings of words in each abstract, excluding common stopwords, to represent the content of each community. Finally, we calculated pairwise cosine similarities between these vectors to measure the textual similarities across communities and identify the range of similarity values.

## Results

### Community Detection and Validation

The Leiden community detection algorithm returned 69 communities with at least 100 papers each. The communities had 25/50/75% size quartiles of [177, 289, 728]. There were 15 communities with at least 1,000 papers. For network validation, across 200 subsamples the network exhibited a high level of consistency in terms of the number of communities (range = [68, 70]), and the organization of specific papers into similar clusters (pair-wise NMI, *M* = .67, median = 0.67, *SD* = .027). NMI is a non-linear measure of the relationship between two random variables (Kachouie & Shutaywi, 2020); an averaged NMI of .6 implies 93% consistency in the clustering solution (Kachouie & Shutaywi, 2020). Thus, the low variation in the number of communities and the high NMI score suggests that the structure of the network is robust to the stochasticity of the community detection algorithm.

### Community Topics and Content

We characterized the main topic(s) of each community by examining the frequency of words from titles, keywords, and abstracts of articles within each community. Table 1 provides concise descriptions of the research topics for each affective science community and serves as a resource for readers to identify specific communities of interest in later sections. It further illustrates the breadth of topics in a significant portion of the affective science field. An inspection of content suggests some broader factors that organize communities such as a focus on certain theoretical constructs, methodologies, populations, and/or applied contexts. We characterized the content of each community using several sources of information, as summarized in the section on “community profiles” below, but provide an overview of the major topic themes here for exposition.

The largest communities focused on broad theoretical constructs in affective science including core affect, appraisal theory, and basic emotions theory (c1), emotion regulation (c2), empathy (c3), and emotional intelligence and emotion labor (c4). Many research communities were also organized by specific emotion category constructs including disgust (c5), fear (c6), shame (c12), happiness (c16), anger (c17), pain (c20), and nostalgia (c59). Certain communities focused on particular modes of expressing affect and emotion including facial expressions (c8, c18), sentiment analysis (c15), expressive writing (c28), and phenomenological analysis (c32). Communities were also centered on individual differences, particularly depression (c41, c42, c45, c64), autism (c50), child development (e.g., c10, c20, c30, c49), and sex or gender (c5, c31, c45, c55). In addition to human populations, some communities were characterized by a focus on non-human animal species (e.g., rodents, c6, c57) or robots and artificial agents (c11, c44, c54). Many of the smaller communities centered on affective science in more specific applied domains including politics (c21, c61), business and marketing (c25, c63), artificial intelligence or gaming (c27, c44), user experience and user interface (c39, c54), tourism and dark tourism (c48, c53), creativity (c43), music (c66), and more.

### Community Interconnectedness

**Fig. 3:**
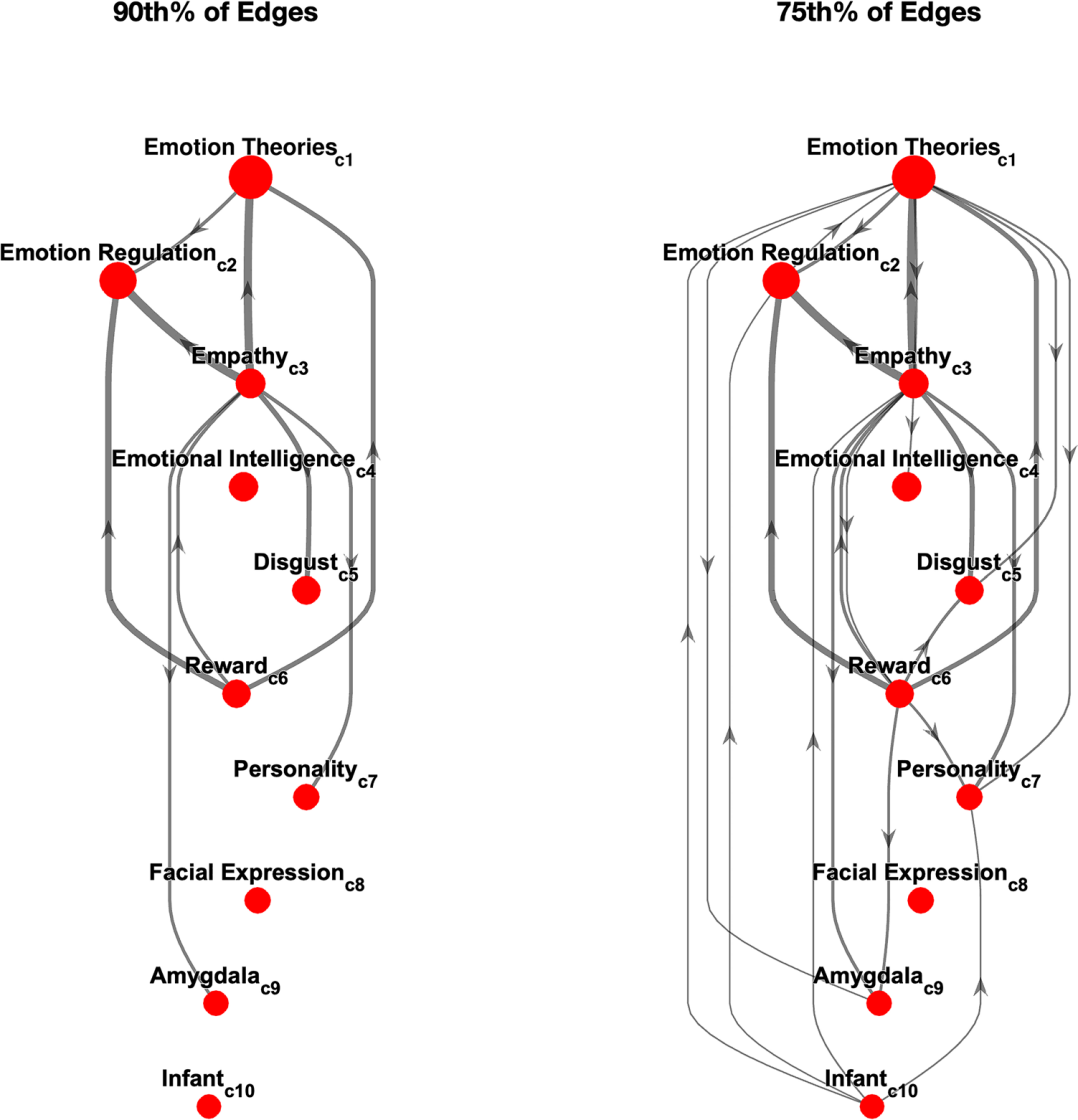
Interconnectedness of the ten largest communities in the affective science network. Interconnectedness was defined as the average shortest path length between nodes of two communities with high betweenness centrality (see Formulas 2 and 3). The left and right plots include interconnected edges between communities that were in the top 90th or 75th percentiles, respectively

Next, we aimed to visualize the interconnectedness of communities in the affective science network. Interconnectedness was estimated using the average of shortest path lengths between nodes with high betweenness values in pairwise communities (see Formulas 2 and 3 above), which normalizes for community size. To create interpretable visualizations of large graphs, we pruned weaker edges and smaller communities. Figure 3 shows interconnectedness between the top 10 largest communities, which comprise over 75% of the papers in our affective science network, for the 90th and 75th percentiles of interconnectedness edges (from the entire, 69 × 69 interconnectedness edge matrix), respectively. Arrows pointing from *c*_i_ to *c*_j_ indicate that *c*_i_ “influences” (i.e., is cited by) *c*_j_.

There are some notable patterns in these visualizations. First, focusing on the 90th% of weighted edges in the entire graph shows that empathy (c3) and reward (c6) are both particularly influential among the largest communities. Specifically, reward (c6) influences the three largest communities including emotion theories (c1), emotion regulation (c2), and empathy (c3) and has no incoming edges. Empathy (c3) influences emotion theories (c1), emotion regulation (c2), disgust (c5), personality (c7), and amygdala (c9) and only has an incoming edge from reward (c6). Second, emotional intelligence (c4), facial expression (c8), and infant (c10) communities were fairly isolated from the other large communities in the top 10. Focusing on the top 75%th percentile of edges still suggests that emotional intelligence (c4) and facial expression (c8) are relatively isolated, while infant (c10) has an influence on several communities (c1, c2, c3, and c8). Overall, these visualizations provide a bird’s eye view of the largest communities and their interrelationships. However, it may also be useful for readers to see which communities have been particularly impactful for, and impacted by, their own communities of interest. We address this point in the community profiles section.

### Identifying Missed Connections

One of our aims in building an affective science network is to identify missed connections between research communities; that is, communities that might benefit from communicating with each other more. We first calculated community distance using our citation-based approach as above. We then estimated content overlap by calculating a “semantic distance” metric, which we operationalized by using embeddings of content words (e.g., from the word clouds) for each community. It stands to reason that communities that cite each other more will also have a lower semantic distance. Indeed, there was a modest but highly reliable correlation of off-diagonal edges across the two distance matrices (Spearman rho = .11, *p* < 1.06e − 12).

Next, we calculated the difference between the *z*-scored edges of each matrix to identify communities that had low semantic distance, but high citation-based distance. The ten pairs with maximal differences in the entire affective science network are presented in Table 2. The table suggests that certain pairs of communities, such as “political” (c21) and “media” (c61), or “socio-political” (c19) and “mind” (c52), or “humor” (c33) and “attitude” (c58), have low citations with each other even though the semantic distance is low. We further present missed connections findings for each community as part of the community profiles below.

**Table 2:** Missed connections: community pairs with high citation-based distance but low semantic distance

From	To	Diff. score	Citation-based distance (*z*)	Semantic distance (*z*)
c21: political	c61: media	3.00	1.46	− 1.54
c19: socio-political	c53: dark tourism	2.85	1.32	− 1.54
c33: humor	c58: attitude	2.77	1.28	− 1.49
c21: political	c58: attitude	2.76	1.46	− 1.30
c39: design	c54: interaction (user)	2.74	1.28	− 1.46
c19: socio-political	c24: self	2.73	1.37	− 1.35
c33: humor	c32: experience	2.71	1.29	− 1.42
c33: humor	c62: culture	2.61	1.28	− 1.33
c33: humor	c28: expressive writing	2.59	1.29	− 1.30
c59: nostalgia	c12: shame	2.58	1.29	− 1.29
c61: media	c58: attitude	2.57	1.29	− 1.28

The table reports the top 10 pairs of communities with the lowest semantic distance, but the highest citation-based distance. We calculated the difference between *z*-scored edges of citation and semantic matrices to identify these community pairs. The table shows that certain pairs of communities, such as “political” (c21) and “media” (c61), or “socio-political” (c19) and “mind” (c52), or “humor” (c33) and “attitude” (c58), have low citations with each other even though they are semantically similar

### Community Profiles

**Fig. 4:**
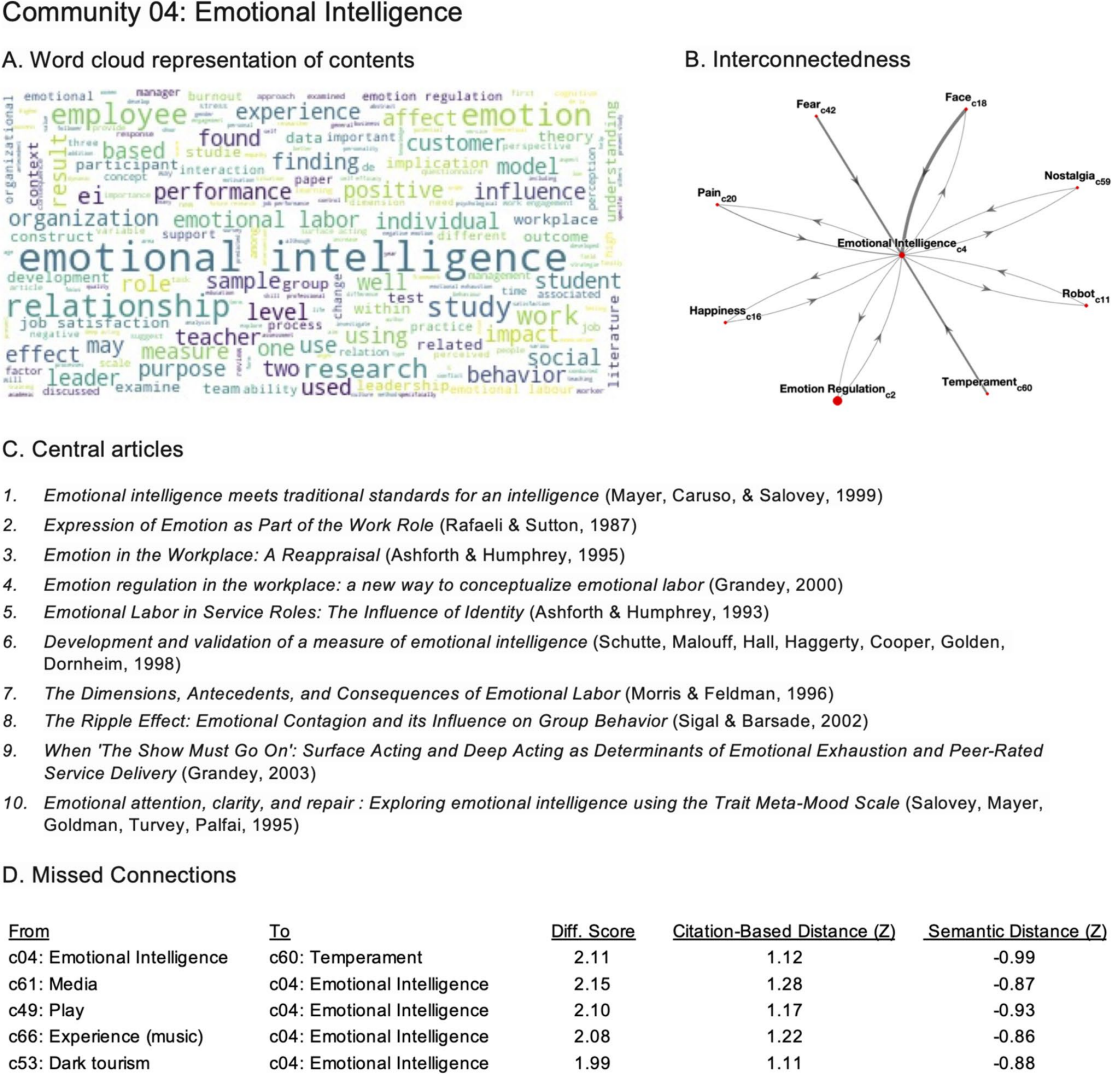
Community profiles. We provide community profiles for all 69 communities on the website Affect Net (https://abs-lab.github.io/affect-net/). The profile for c4 is presented as an example. Community content can be discerned using both **A** a Wordcloud of terms from the titles, abstracts, and keywords of articles in c4, and also **C** a list of the ten articles in the community with the highest outdegree centrality scores. **B** The profile also shows which communities are most interconnected with c4, the directionality of influence, and **D** potential missed connections of c4 with other communities (see main text for a more detailed discussion of c4’s profile)

Perhaps for most scientists, it will be of particular interest to zoom in on particular research communities from Table 1. Thus, for each community, we created a profile that provides an organized representation of content, interconnectedness, and missed connections. As an example, we provide the community profile for c4, on “emotional intelligence” in Fig. 4. The profile provides both low- and high-dimensional representations of content from keywords, a Wordcloud, and the titles of ten articles with the highest outdegree centrality, clearly implicating the community in emotional intelligence and how it pertains to labor, education, the workplace, etc.

For interconnectedness, we included the top four most proximal communities that either cite or are cited by c4. The same community could be—and often was—in the top four for both cite, or cited by, interrelationships with c4. As shown in Fig. 4B, c4 on emotional intelligence had reciprocal influences on several communities including emotion regulation, happiness, pain, nostalgia, and robot. It appears to be relatively more influenced by c18 on facial expressions than vice versa, and also influenced by c42 on fear, and c60 on temperament. Further inspection of these community contents using the website indicates that many of the communities with interconnections with c4 include developmental keywords (e.g., “infant, children, adolescent, parent, development, attachment”). Finally, there are several potential missed connections for c4 (Fig. 4D). In particular, c40 on language, c61 on media, c49 on play, c66 on experience (music), and c53 on dark tourism, all have high citation-based distance but low semantic distance, suggesting that there may be opportunities to forge theoretical or empirical bridges between these communities. Readers can explore the community profiles for all 69 communities on the Affect-Net website.

## Discussion

As the scientific field matures, there is increasing research volume, specialization, and siloing into communities (Bornmann & Mutz, 2015; Morillo et al., 2003; Van Noorden, 2015; Wang & Wang, 2020). Each generation of scholars has an exponentially larger volume of research to contend with while simultaneously mastering the methodological skills of their area of specialization which is also becoming increasingly sophisticated (Altman & Cohen, 2022; de Meis & Leta, 1997; Luke et al., 2015). Correspondingly, contemporary scientists face unique challenges in acquiring both breadth and depth of scholarship. Such scholarship is crucial for generating new scientific questions in the field, developing interdisciplinary theoretical frameworks, and learning from both the successes and failures of past work.

Our aim was to provide scientists with a compact, data-driven map of affective science that can be used to facilitate intercommunication between research communities and to guide the acquisition of breadth and depth of knowledge in the field. Here, we summarized the contents of over one hundred thousand articles, originating since the very beginnings of affective science, into a set of 69 communities. A brief reading of Table 1 and a perusal of community profiles can provide established scientists and trainees alike with an efficient overview of a substantial portion of the field in an astoundingly brief amount of time. In our discussion below, we evaluate the utility of this data-driven approach to modeling a field and as a resource for the affective science and broader scientific community.

### How Many Research Communities Comprise the Affective Sciences Field?

While it is well-acknowledged that affective science is undergoing an explosion of research, it remains unclear as to how many research communities there are and the scientific topics they span. Our CNA suggests there are at least 69 citation-based research communities in the affective science network (Table 1). Of note, we used a data-driven, stochastic clustering approach to identify these research communities. The clustering algorithm will generate as many clusters as are justifiable, given the data. In our case, we submitted a large network of over 100 thousand papers to the clustering algorithm to generate the broadest characterization of communities in the affective science field. For tractability, we also only discussed communities with at least 100 research papers. Accordingly, the 69 communities we list should be thought of as, at most, a lower bound for the number of communities in the affective science literature. Importantly, CNA could also be iteratively performed on papers from each of the communities we observed here. This strategy may reveal a finer-grained structure within each community, which is likely to be a useful direction for future work toward developing focused reviews of specific research areas.

Finally, it is important to note that the choice of community detection algorithm can influence the number and structure of the resulting communities (Danon et al., 2005; Yang et al., 2016). For example, switching between different algorithm families (e.g., modularity-based like Leiden versus flow-based like Infomap) may yield significantly different community structures. We selected the Leiden algorithm for its efficiency in handling large networks, its ability to produce well-connected and stable communities, and its optimization of modularity while preventing disconnected communities. These features make it ideal for our large-scale citation network, balancing computational speed with community detection accuracy.

### What Are the Contents of Research Communities?

Communities in affective science were organized around theoretical constructs, methodologies, populations, and applied contexts (Table 1 and as derived from individual community profiles). The four largest communities focused on broad emotion theories, emotion regulation, empathy, and emotional intelligence. There were also communities centered on specific emotion categories, modes of affect expression (e.g., facial expressions, language), individual differences, subject populations, and non-human animals or artificial agents. Smaller communities concentrated on applied domains such as politics, artificial intelligence, gaming and user experience, tourism/dark tourism, creativity, music, and pets. Major families of emotion theories such as appraisal theory, basic emotions theory, or core affect theory, were represented across many communities reflecting their broad influence on affective science subfields. The largest community, “Emotion Theory,” encompasses most of the literature on emotion theory, with its top three most relevant papers showcasing diverse theoretical perspectives from Ekman (1992), Scherer (2005), and Russell (2003).

We also provide a multidimensional look at community content, which can be useful for identifying how communities with certain shared topics also diverge from one another. For instance, both c41 and c64 emphasize depression, yet the major concepts of c41 also include treatment and behavior, whereas c64 emphasizes disease and illness. Similarly, the content analyses revealed communities that share an interest in faces but form distinct citation-based communities including the largest community, c1, which likely concerns the role of faces according to core affect and basic emotions accounts, but also other communities that focus on recognition and categorization of facial expression (c8), hemispheric lateralization in face processing (c18), smiles (c34), and crying (c51).

In addition to providing an overview of many of the research communities, the content analysis also lends insight into whether and how the ontology of affective science (e.g., see (Barrett, 2009; Hastings et al., 2012; Scarantino, 2012) relates to the community structure. An ontology refers to the concepts that are presupposed by a field and how they relate to one another (Gruber, 1993; Lacasta et al., 2010). It is interesting to consider that the citation-based identification of research communities need not recapitulate the major ontological concepts in affective science, and yet, they seemed to. Indeed, many of the larger and medium-sized communities focus on core affect and several specific emotion categories, as well as emotion regulation, empathy, and other major ontological concepts in the field. As (Barrett, 2022; Dubova & Goldstone, 2023) note, a field’s ontological structure can have a profound impact on shaping scientific truths in affective science. If not careful, this can lead to circular reasoning wherein the scientific methodology artificially creates findings that are hypothesis-consistent, rather than providing rigorous tests of these hypotheses. In affective science, a key debate (as reflected in the largest community, c1) concerns whether the ontological concepts pertaining to emotion categories, such as disgust, fear, and joy, are “carved in nature” (Barrett, 2006; Ekman, 1992; Ortony & Turner, 1990; Russell, 2009; Scarantino & Griffiths, 2011). Evidence that instances of the same category (e.g., of fear) are more similar to one another, and more distinct from instances of other categories (e.g., of anger, disgust), is often interpreted as favoring a biologically basic view of emotion. However, the methods used to generate data are already predisposed to create categories even if, in reality, evidence of a categorical structure did not exist (for a detailed argument, see (Barrett, 2022). The finding that many research communities are focused on particular emotion categories suggests that ontologies may not only guide theory and methods in affective science but also how research communities are formed and organized, suggesting that even more caution must be taken to avoid substituting ontological assumptions for scientific facts about the nature of emotion.

Ontologies may also change over time with paradigmatic shifts in the field. For example, the National Institute of Health’s Research Domain Criteria (RDoC) is an explicit reorganization of the classical ontology of psychiatric categories (depression, anxiety, schizophrenia, etc.) that instead places more emphasis on the psychological mechanisms that are shared across individuals even with distinct diagnoses (Bluhm, 2017; Carpenter, 2016; MacNeill et al., 2021). Intriguingly, our analysis suggests that communities were not strongly organized by specific psychiatric categories. The most extreme examples were c41 and c64, both of which emphasized depression, but which also contained content pertaining to other psychiatric categories.

As Knobe (2007) discusses in the context of experimental philosophy, the methods used to collect data can shape the conclusions drawn, thus we need to exercise caution when interpreting the roots of community formation. Indeed, scientific communities can be shaped not only by ontological assumptions but also by social and methodological influences (Bird, 2013; Carnap, 1950; Leonelli, 2016).

### Which Research Communities Influence Each Other and Which Might Have Missed Connections?

A third aim of our CNA was to identify which communities influence each other and which might benefit from further communication. Interconnectedness was examined among the top 10 communities which contain 75% of the papers in our network and by displaying the most influential interconnections for each community in their respective profiles. We also identified potential missed connections between communities, which are listed in Table 2 for the maximal cases throughout the network and also for each community in their respective profiles. In doing so, we compared and contrasted citation-based distance with semantic distance. Intriguingly, semantic distance only weakly predicted citation-based distance. This low correlation suggests that the citation-based distance between communities is unlikely to be driven by simple semantic overlap. A comparison of these distance metrics also highlighted potential missed connections between communities, offering insights rather than definitive conclusions about the presence or absence of such connections. These measures of interconnectedness and missed connections may be most valuable for readers focused on specific communities. For instance, focusing on emotional intelligence (c4) shows that while it was isolated from several of the other large communities (with the exception of emotion regulation), it was influenced by (i.e., cited) several other communities that emphasized certain affective categories (positive emotions, fear, and pain), face processing and research communities on development and temperament. It also has potential missed connections with certain communities including those that focused on play, media, and music experience, suggesting new directions for interdisciplinary work that connects emotional intelligence with these communities. As another example, the largest community focusing on emotion regulation (c2) was influenced by several communities on emotional development in children, emotional intelligence, reward, and expression recognition, which may provide scholarly and historical insight into how this very large area of work in affective science developed. In many if not most cases, the influences of communities upon one another were bidirectional. Such bidirectionality may also depend on time since the overwhelming majority of publications in affective science have occurred within a relatively short time span over the past two decades (Fig. 1B), a period when research communities likely evolved concurrently. Here, our main goal was to provide readers with the tools to examine the influences and missed connections of their research communities of interest. However, we recognize that identifying missed connections between communities may not be sufficient to address the roots of divisions, especially when theoretical frameworks are incompatible or opposing. While our analysis can help identify potential areas for collaboration, it is important to acknowledge that deeper philosophical and theoretical discussions may be required to reconcile such divides and facilitate meaningful collaboration between communities. Thus, while our data-driven approach highlights opportunities for increased interaction, bridging these gaps often necessitates addressing the foundational issues that create barriers between communities.

### Citation Timelines and History of the Affective Science Field

Our CNA, which provides a static snapshot of the affective sciences field, may underrepresent younger and emerging subfields or interdisciplinary areas, such as political science, sociology, and anthropology, which may not yet form densely cited groups. These communities, while ontologically meaningful, may not have accumulated enough publications or a web of citations to be detected as distinct clusters. Furthermore, the timing of data collection significantly affects the results, as fast-moving fields may not be fully captured in a static analysis. A clear future direction is to examine how each of these communities formed and changed over time. We opted to limit our scope in the present paper for certain reasons. The mathematical modeling of dynamic graphs is itself an emerging area of work in network science. Furthermore, dynamic graph analysis also leads to many novel research questions regarding the evolution of a scientific field, and perhaps even forecasts of the future, that are better addressed in a separate work.

Still, our current CNA can provide some initial insights on time, at least pertaining to the influences of certain communities over time. For instance, each community profile includes the publication years of its most central articles. A perusal of interconnectedness graphs also suggests that certain communities, such as c42, have had a consistent, unidirectional influence on numerous other communities. Many of c42’s central articles were published in the 1960s and 1970s, during which the field was still quite small in volume. The contents of c42 suggest that it focused on attachment theory and non-human primate models of anxiety and depression. Notably, c42 is not a particularly large community in our network, but it appears to have a considerable impact on the affective sciences field as a whole. These initial observations based on our current CNA suggest that more formal dynamic analyses of citation networks are likely to be an exciting direction for future work.

### Accelerating Scholarship and Enabling Interdisciplinary Research

CNA can serve as a powerful tool for scholars to orient themselves in a vast field and develop their scholarship (Colicchia et al., 2018). With the growing volume of published literature, it can be daunting for scientists to develop confidence in their scholarship of a field as vast and heterogeneous as affective science. Here, our implementation of CNA and content analysis can be a useful resource for navigating the literature. Our map of the affective science network may help “level the playing field” by providing scholars throughout the world, regardless of their opportunities for mentorship, with the means to approach the vast amount of literature in the field (McLaren & Bruner, 2022). In other words, we provide an additional tool that can support trainees—especially those who lack access to mentors or academic resources—by helping them understand the structure of the field and explore the literature more effectively. To be sure, in the ideal case, our map offers a complementary resource for both trainees and also mentors, such that the trainee benefits from multiple sources of input to guide scholarship development in the field.

This map may also be used to identify and quickly learn about research communities that are adjacent to but related to one’s own, with the potential to accelerate interdisciplinary collaboration.

## Conclusions and Future Directions

The explosion of research in affective science has led to unique new challenges for interdisciplinary research, scholarship development, and tracking the trajectory and history of the field. Conventional approaches to addressing these issues are not equipped to handle this massive and accelerating volume of research. Here, we develop the first, field-wide network of the affective sciences literature. We identified dozens of research communities and characterized their contents, interrelationships, and potential missed connections. This map of the field provides interpretable and accessible, low-dimensional representations of well over a hundred thousand articles. Information about affective science communities is easily accessible on the accompanying website “Affect-Net,” which may be useful for established scientists, and also students or trainees, to guide scholarship development and provide inspiration for new interdisciplinary directions. Future work may build on our affective science network by tracking the development of communities over time and by using approaches that integrate multiple sources of information (e.g., authors, publication years, text, and meaning analysis) when building network maps of the field.

## Additional Information

### Data Availability

Data will be provided via a Dropbox folder, upon acceptance. The scripts of the analysis will be publicly available on our Github, upon acceptance.
