# Correction: The Affective Science Network: A Fieldwide Map of over 1 Million Citations

**DOI:** 10.1007/s42761-025-00299-9

**Published:** 2025-03-04

**Authors:** Alessia Iancarelli, Nicholas R. Rypkema, Maureen Ritchey, Ajay B. Satpute

**Affiliations:** 1 Department of Psychology, Northeastern University, 360 Huntington Ave, 125 NI, Boston, MA 02115 USA; 2 Applied Ocean Physics & Engineering, Woods Hole Oceanographic Institution, Woods Hole Rd, Woods Hole, MA 02543 USA; 3 Department of Psychology and Neuroscience, Boston College, Chestnut Hill, MA 02467 USA; 4 Department of Psychology, Core Faculty, Institute for Experiential Artificial Intelligence, Northeastern University, Boston, MA 02115 USA; 5 Department of Radiology, Massachusetts General Hospital, Boston, MA 02114 USA

**Correction: Affective Science**

10.1007/s42761-024-00292-8

The author found out that there is an error in the below reference that needs to be corrected.

Correct citation:

Becker, A., & Lukka, K. (2023). Instrumentalism and the publish-or-perish regime. Critical Perspectives on Accounting, 94, 102436. 10.1016/j.cpa.2022.102436.

Incorrect citation in the paper:

Ioannidis, J. P., Becker, A., & Lukka, K. (2022). Instrumentalism and the publish-or-perish regime. Epidemiology, 94, 102436.

This erratum is presented to fix these errors.

The original article has been corrected.

